# New Evidences about Subjective Well-Being in Adolescence and Its Links with Neurocognitive Performance

**DOI:** 10.3390/ijerph17061866

**Published:** 2020-03-13

**Authors:** Javier Ortuño-Sierra, Rebeca Aritio-Solana, Eduardo Fonseca-Pedrero

**Affiliations:** 1 Department of Educational Sciences, University of La Rioja, 26002 Logroño, Spain; rebeca.aritio@unirioja.es (R.A.-S.); eduardo.fonseca@unirioja.es (E.F.-P.); 2 Centro de Investigación en Biomédica en Red de Salud Mental (CIBERSAM), 33005 Oviedo, Spain

**Keywords:** satisfaction with life scale, wellbeing, neurocognitive, mental health, adolescence

## Abstract

The main purpose of the present work was to study the neurocognitive endophenotypes of adolescents at risk for low personal wellbeing. The sample included a total of 1588 adolescents from stratified random cluster sampling; derived from this sample, a group of high-risk (*n* = 84) and a control group (*n* = 84) were selected. The personal well-being index–school children (PWI–SC), the University of Pennsylvania computerized neuropsychological test battery for children (included 14 tasks assessing five neurobehavioral domains: executive functions, episodic memory, complex cognition, social cognition and sensorimotor speed), and the strengths and difficulties questionnaire (SDQ) were used. Adolescents with low personal wellbeing showed statistically significant impairments across the different neurocognitive domains. In particular, adolescents at risk showed lower accuracy scores on executive function and complex cognition and lower speed scores on episodic memory, complex cognition and social cognition scores. The results of the present study contribute relevant information about the nature of neurocognitive impairments associated with subjective wellbeing and allow implementing preventive treatments.

## 1. Introduction

Adolescence is considered a crucial developmental stage with different transformations that impact physical, psychological and sociological levels [1]. Related to these changes, different authors have revealed that there is an increase in psychological difficulties ranging from transient mild symptoms to full-blown anxiety disorders [2]. For instance, prevalence rates of depression and anxiety increase at this time [3,4], and it has also been documented that adolescents are at a higher risk of emotional and behavioral difficulties [5]. With this regard, the study of personal wellbeing during adolescence, in addition to other psychological measures, could be a key indicator of mental health and related phenomena [6].

Considering the high prevalence and the long-term negative consequences associated with mental health difficulties during adolescence, more attention and resources are now being devoted from public health systems to the evaluation, detection and intervention of psychological difficulties [7,8]. In addition, the study of protective and risk factors, for instance, neurocognitive performance is recommended, in order to better understand those individuals at risk for psychological difficulties [9]. Moreover, identifying specific neurocognitive factors in young people at high-risk may allow the optimization of the prognostic accuracy and the prediction strategies of clinical outcomes [10]. The study of biobehavioral indicators and its linkage to brain functioning could help us to establish developmental pathways of cognitive development related to subjective wellbeing and mental health impairments. Previous literature suggests the links between cognitive and neurobehavioral performance and emotion domains, although, to date, there is a lack of literature analyzing these connections, including the manifestation of wellbeing [11,12].

Subjective wellbeing (SWB) is understood as a conscious cognitive judgment of life in which individuals compare their life circumstances with a self-imposed standard [13]. In order to indicate the level of SWB, the terms of positive and negative affect are used. In this regard, people who have experienced more positive affect than negative affect are considered as having higher SWB [14]. Recent studies highlight the relationship of SWB with socioeconomic aspects and quality of life [15]. In addition, SWB and youth happiness have been related to different psychological difficulties and mental health, and linked wellbeing in adolescence with adult life satisfaction [16,17]. Thus, it seems that studying the subjective wellbeing of children and adolescents could be relevant, considering the consequences of this particular time in which adolescents establish personality and life-course trajectories of health and wellbeing [18]. For instance, different studies have shown that health and behavior patterns that became permanent throughout adulthood were related to adolescence levels of subjective wellbeing [19,20].

A recent study by Tickell et al. [21] revealed different profiles of young inpatient adults relating to neurocognitive performance. Neurocognitive domains such as sustained attention or memory were related to anxiety and stress, among others. In another relevant study with young people between 17 and 24 years old, Morey-Nase et al. [22] found that major depression was related to subjective experiences of neurocognitive functioning.

The knowledge of how emotional difficulties, and subjective wellbeing in particular, are related to neurocognitive performance has clear implications in order to understand mechanisms underlying different neurocognitive impairments associated to psychological difficulties. The evaluation of difficulties in subjective wellbeing can also allow us to act before these difficulties become more severe and, therefore, to prevent possible future mental health problems. Early detection strategies could allow us to implement prophylactic interventions in a specific developmental stage like adolescence, where changes may have a significant impact [23]. Considering this previous background, the main goal of the present study was, therefore, to characterize the neurocognitive phenotype of adolescents at risk for low wellbeing by comparing them with the age–gender-matched comparison group. Based on previous literature, we hypothesized that those at high risk for low levels of psychological wellbeing would show deficits that would be more robust across neurocognitive domains compared to those adolescents at nonrisk.

## 2. Method

### 2.1. Participants

A stratified random cluster sampling was conducted at the classroom level in a population of approximately 15,000 students from La Rioja (a region located in northern Spain). The students belonged to different types of public and concerted educational centers of compulsory secondary education and vocational training, as well as to different socioeconomic levels. With the aim to establish the stratus, we considered the type of school (public/private) and the scholar stage (compulsory stage, post-compulsory and professional formation), where the probability of classroom extraction was determined as a function of the total number of students.

The initial sample was composed of 1881 students. Those participants who presented: (a) a high score on the Oviedo infrequency response scale (more than 3 points; *n* = 104), (b) an age older than 19 (*n* = 170), or (c) not completing the test (*n* = 76) were eliminated. The final sample comprised 1588 students, 739 males (46.5%) and 849 (53.5%) females, belonging to 34 schools and 98 classrooms. The mean age was 16.13 years (SD = 1.36), ranging from age 14 to 19 years (14 years, *n* = 213; 15 years, *n* = 337; 16 years, *n* = 400; 17 years, *n* = 382; 18 years, *n* = 180; 19 years, *n* = 76). The nationality distribution was as follows: 89.9% Spanish, 3.7% Latin American (Bolivia, Argentina, Colombia and Ecuador), 0.7% Portuguese, 2.4% Romanian, 1% Moroccan, 0.7% Pakistani and 2% other nationalities.

With the aim to compare at risk and nonrisk adolescents for low subjective wellbeing, two different groups were established. For the psychometric risk group, based on previous research [24], scores lower than 43 were considered as an indicator for the inclusion criteria for the PWI–SC. A total of 106 adolescents were selected based on this criterion. Penn computerized neurocognitive battery (CNB) z-scores lower than −3 and higher than 3 were eliminated from the sample in order to prevent the influence of outliers. Thus, a final sample of *n* = 84 (43 males, mean age = 15.88 years old) was included in the high-risk group. In order to establish an age and gender matched group, individuals were selected based on the gender and age parameters of the risk group; thus, the final sample of the comparison group was comprised of *n* = 84 (43 males, mean age = 16.11 years old).

### 2.2. Instruments

The Penn computerized neurocognitive battery (CNB) [25,26] is a single computerized battery that combines tests from multiple batteries, which is one of its main strengths. As proposed by Gur et al. [26] instructions and vocabulary for verbal stimuli were simplified from the adult CNB. The CNB was developed with the aim to support large-scale genomic studies. CNB was administered to participants using a system developed at the University of Pennsylvania (US). The 1-h computerized neurocognitive battery included 14 tasks assessing five different domains: executive functions (abstraction and mental flexibility, attention and working memory), episodic memory (words, faces and shapes), complex cognition (verbal reasoning, nonverbal reasoning and spatial processing), social cognition (emotion identification, emotion intensity differentiation and age differentiation) and sensorimotor speed (motor and sensorimotor).

The CNB includes different neurobehavioral indicators with different tasks adapted to guarantee psychometric properties and their linkage to brain systems for children [27]. Moreover, previous studies have shown adequate psychometric properties [25,27]. In particular psychometric properties with adolescent population have been confirmed [28]. Except for the tests designed exclusively for measuring speed, each test provides measures of both accuracy and speed. According to previous research [25,28], the web-based platform for the CNB was developed using Perl CGI, HTML, a mySQL database and the Apache webserver; tests were developed using Adobe Flash^®^, and scoring was fully automated.

The personal well-being index–school version (PWI–SC) [29] is derived from the personal well-being index for the adult population (PWI–A) [30]. The PWI–SC contains seven items of satisfaction corresponding to the different quality of life domains: standard of living, health, life achievement, personal relationships, personal safety, community-connectedness, future security and spirituality–religion. Items of the PWI–SC are presented in a ten-option Likert response format ranging from 0 = very unsatisfied to 10 = very satisfied. The score on the global scale is the result of adding up the scores on these seven items; therefore, the total score can range from 0 to 70 points. A higher score indicates higher levels of subjective wellbeing. The PWI–SC was modified in order to make the wording more accessible for children and adolescents. In addition, satisfaction was substituted by happiness. The psychometric properties of the instrument have been confirmed in previous studies [31]. The PWI–SC has been validated in Spanish samples of adolescents [24].

The family affluence scale-II (FAS-II) [32] was used to measure socioeconomic status using a four-item child-appropriate measure of family wealth with score ranges from 0 to 9. A higher score indicates higher levels of socioeconomic status. Previous international studies have demonstrated its adequate psychometric properties [32].

The strengths and difficulties questionnaire (SDQ) [33] self-reported form was developed with the intention to measure emotional and behavioral problems. The SDQ comprises a total of 25 items distributed in five subscales. The different subscales were: emotional symptoms, conduct problems, hyperactivity, peer problems and prosocial behavior. The SDQ items are presented in a three-option Likert response format (not true = 0, somewhat true = 1, certainly true = 2). Consequently, the score on each subscale goes from 0 to 10 points. The sum of the difficulty’s subscales (all of them besides the prosocial behavior) displays the total difficulties score that ranges from 0 to 40 (a higher score reveals more difficulties). The Spanish version (www.sdqinfo.org) of the SDQ, validated in previous studies [34] was used.

The Oviedo infrequency scale (INF-OV) [35] is an instrument developed to determine participants responding in a dishonest manner. The INF-OV contains a total of 12 items with a 5-point Likert scale format (1 = completely disagree; 5 = completely agree). The guidelines for test construction were used to develop the instrument; an example of items of the INF-OV is: “I know someone that wears glasses.” Those students that reveal three or more incorrect responses are eliminated from the sample. This measuring instrument has been administered in previous research [35].

### 2.3. Procedure

The research was approved by the Department of Education of the Government of La Rioja and the Ethical Committee of Clinical Research of La Rioja (CEImLAR, CEImLAR P.I. 337).

The CNB and self-reports were administered by previously trained researchers and followed a standard protocol. Thus, those CNB tasks requiring a greater cognitive effort (e.g., matrix reasoning) were either preceded or followed either by a break or a test involving motor speed (e.g., finger tapping). The CNB order and some other conditions created to prevent fatigue and frustration were followed, based on Gur et al. [25] recommendations, and participants were offered breaks approximately every 20 min. Both measures were administered collectively, in groups of 10 to 30 students, during regular school hours, and in a classroom especially prepared for this purpose. Participants were informed about the voluntary nature of the study and no incentive was provided for participation. Parents or legal tutors gave informed consent for participants under 18 years old.

### 2.4. Data Analyses

Raw scores for accuracy and speed for each test were calculated and converted, then z-transformed to their standard equivalents attending to means and standard deviations for the entire sample. Higher z-scores always were intended to reflect better performance (i.e., higher accuracy and shorter responses correspond to higher z-scores) so as to facilitate interpretation and to make it consistent; thus, response time z-scores were multiplied by −1, so that slower response time was reflected in lower z-scores.

Descriptive statistics for accuracy, speed and efficiency measures of the five neurobehavioral domains of the CNB were calculated based on at-risk and nonrisk for low wellbeing. Second, a MANCOVA was performed taking the four neurocognitive domains (executive function, memory, complex cognition, social cognition and sensorimotor in the case of speed) as the dependant variables and the two groups derived from the PWI–SC scores (at-risk vs. nonrisk) as the fixed factors. For the analysis, socioeconomic status and the SDQ total difficulties score were controlled as potential covariates affecting the results. Partial eta squared (*partial* η^2^) was employed as an effect-size estimate. SPSS 22.0 was used for data analyses [36].

## 3. Results

### 3.1. Descriptive Statistics for the Neurocognitive Domains by groups

A total of 168 participants from the community-derived sample of adolescents were selected as at-risk (*n* = 84) and nonrisk (*n* = 84) based on the PWI–SC. Descriptive statistics for all the *z*-scores in the neurobehavioral functions based on accuracy, speed and efficiency are shown in Table 1.

### 3.2. Accuracy Performance across Neurocognitive Domains by groups

After controlling for the effects of the participant’s socioeconomic level and psychological difficulties, results of the MANCOVA, with the accuracy scores of neurocognitive domains as dependent variables and the two groups (at-risk vs. nonrisk) as fixed factor, revealed a main effect for group (*λ* = 0.917, *F*_(4,155,000)_ = 2.99, *p* ≤ 0.05, *partial η*^2^ = 0.059) (see Table 2).

The ANOVAs revealed statistically significant differences by group in executive control (*F*_(1,157)_ = 7.855, *p* ≤ 0.05, *partial η*^2^ = 0.037) and complex cognition (*F*_(1,157)_ = 7.017, *p* ≤ 0.05, *partial η*^2^ = 0.041), but not for the other two: memory (*F*_(1,157)_ = 1.109, *p* ≥ 0.05, *partial η*^2^ = 0.003) and social cognition (*F*_(1,157)_ = 0.014, *p* ≥ 0.05, *partial η*^2^ = 0.001). The effect sizes found were low. Youths at risk for low wellbeing showed a significant decrease in performance accuracy across these neurocognitive domains compared to nonrisk.

### 3.3. Speed Performance across Neurocognitive Domains

The MANCOVA on speed scores revealed a main effect for group (*λ* = 0.912, *F*_(4,152,000)_ = 2.753, *p* ≤ 0.05, *partial η*^2^ = 0.081). As shown in Table 3, the ANOVAs indicated that participants’ speed scores in the five different domains differed significantly according to the group in episodic memory (*F*_(1,153)_ = 5.316, *p* ≤ 0.05, *partial η*^2^ = 0.032), complex cognition (*F*_(1,153)_ = 10.501, *p* ≤ 0.05, *partial η*^2^ = 0.069) and social cognition (*F*_(1,153)_ = 7.505, *p* ≤ 0.05, *partial η*^2^ = 0.052). However, no statistically significant differences were found for the other domains: executive control (*F*_(1,153)_ = 0.409, *p* ≥ 0.05, *partial η*^2^ = 0.03) and Sensorimotor domain (*F*_(1,153)_ = 0.247, *p* ≥ 0.05, *partial η*^2^ = 0.002). Therefore, youths at-risk showed a significant decrease in speed performance (slower time) in episodic memory, complex cognition, and social cognition when compared to the nonrisk group.

### 3.4. Efficiency Performance across Neurocognitive Domains by groups

The study of the efficiency (means values of accuracy and speed scores) in the four domains, showed a main effect for group (*λ* = 0.729, *F*_(4,150,000)_ = 8.035, *p* ≤ 0.001, *partial η*^2^ = 0.111) in the MANCOVA scores. Results from the ANOVAs are shown in Table 4. As can be seen, adolescents of both groups differed significantly in efficiency performance scores in executive control (*F*_(1,151)_ = 9.025, *p* ≤ 0.05, *partial η*^2^ = 0.061), episodic memory (*F*_(1,151)_ = 6.205, *p* ≤ 0.05, *partial η*^2^ = 0.016), complex cognition (*F*_(1,151)_ = 12.013, *p* ≤ 0.05, *partial η*^2^ = 0.072) and social cognition domains (*F*_(1,151)_ = 3.428, *p* ≤ 0.05, *partial η*^2^ = 0.018). Individuals at-risk for low subjective wellbeing showed a significant decrease in efficiency scores across all neurocognitive domains when compared to the nonrisk group.

## 4. Discussion

The main goal of this study was to analyze the association between neurocognitive domains and subjective wellbeing. Based on previous studies, we hypothesized that psychological wellbeing would be related to neurocognitive performance. The results confirm our hypothesis, revealing that subjective wellbeing was associated with different neurocognitive domains (e.g., executive control, episodic memory, complex cognition and social cognition) in adolescents. Specifically, those individuals with a poorer perception of their personal wellbeing showed a statistically significant lower neurocognitive performance than those with higher levels of subjective wellbeing.

Adolescence is a critical developmental stage for the onset of psychological difficulties and mental health problems, ranging from transient mild symptoms to full-blown anxiety disorders [2]. The core challenge in this age span is the derivation of developmentally more sensitive assessment methods. Identification of characteristics that could serve as solid predictors for onset, course and outcome requires prospective designs that assess a wide range of putative vulnerability, as well as protective and risk factors. Results found in this study have clear implications for research, educational and clinical settings. The comprehension of how subjective wellbeing is related to different neurocognitive measures may allow the implementation of early prevention strategies in specific settings such as school. Screening for psychological wellbeing and implementing educational and psychological programs to promote subjective wellbeing at schools could prevent short and long-term difficulties in neurocognitive performance.

We studied the neurobehavioral performing of the adolescents based on the accuracy, the speed and the efficiency of their scores. The analysis of the accuracy reflected that adolescents at risk for low personal wellbeing had a statistically significant lower performance on episodic memory and social cognition compared to adolescents without risk. Previous research has shown that individuals with psychological difficulties such as suicide problems have worse scores in tasks related to memory [37] and recognition of emotion and social signals [38,39]. As has been suggested, it is possible that the diminished perception of quality of life is linked to circuitry and neurochemistry abnormalities, including impairments of the serotonin neurotransmitter system and the hypothalamic–pituitary–adrenal axis stress–response system that lead to impairments such as over-reactivity to negative social signs [40]. Adolescence is a critical stage in which cognitive function is still developing and, based on epigenetic mechanism psychological difficulties, could be interconnected with emotional recognition and deficits in the recruitment of the attentional control neural circuitry, when related to attention to mild negative stimuli [38].

When analyzing adolescent speed performance, the results showed that adolescents at risk for low personal wellbeing had lower scores on complex cognition compared to the nonrisk group. Previous literature revealed that problem-solving abilities [41,42], or cognitive inhibition and interference management capability [43,44] was reduced in individuals with mental health problems, including suicidal behavior. In this sense, the results found in the present study seem to confirm previous findings.

The neurocognitive performance as a result of speed and accuracy was also studied by means of adolescents’ efficiency. Statistically significant differences between the risk and the nonrisk groups were found in all the domains. These results are consistent with the idea that adolescents with different psychological difficulties including internalizing and externalizing problems or suicide problems have impairments in selective attention, cognitive flexibility, emotional recognition or emotional processing [38,39,45,46]. Thus, the results confirm the relationship between neuropsychological functioning and wellbeing, with phenotypic differences between groups of risk and nonrisk adolescents.

The knowledge of this evidence is relevant in order to allow for the early identification of those at-risk for poor wellbeing in community or educational settings. In addition, it allows for the implementation of close-in strategies or a two-stage process model in order to further comprehensively evaluate mental state or early interventions to improve the outcome. Different studies have shown the relationship between cognitive functions and mental difficulties with related brain areas [40,46]. Nonetheless, there is a lack of studies analyzing the performance in different neurocognitive domains and its relationship with subjective wellbeing. Results from studies analyzing genomic and neuroimaging have pointed out the relevance of neurobiological screening in order to prevent mental health problems [40].

A relevant number of children and adolescents would potentially present mental health difficulties throughout their life, having a clear impact not only on their personal lives but also on health and economic levels [47,48,49]. Impairments in neurocognitive domains have been found to be linked with psychological issues indicating a potential common underlying neurodevelopmental disorder, as well as links to cognitive functioning [40,46,50,51]. In particular, previous research analyzing more severe mental health problems and neurocognitive functioning are in line with the results found in this study. For instance, Tickell et al. [21] found that impairments in different neurocognitive domains were related to anxiety and stress, among others. In addition, a recent study revealed the association between neurocognitive functioning and depressive symptoms [22].

Considering these recent studies, the present study seems to have relevant implications for clinical settings. Difficulties in wellbeing are associated with psychological difficulties that may lead to serious mental health problems [52]; therefore, the evaluation of alarm signals of difficulties in subjective wellbeing can allow us to detect earlier and prevent possible future mental health problems. Strategies aiming to understand and detect cognitive endophenotypes are relevant in order to implement prevention and early detection strategies with therapeutic targets in the mental health field [53]. Thus, designing emotional wellbeing programs with the purpose to promote social and emotional skills in different settings, including school, seem necessary. Focusing on early prevention programs instead of palliative interventions is always the most thorough response.

This study had some limitations. One possible limitation is that we focused on a particular Spanish region located in the north; future studies could expand the analysis to other regions of the country. With this regard, cross-cultural studies, including different countries, would also be relevant. There is also an inherent issue in the administration of every type of self-reported instrument, with the very well-known effect of stigmatization, the possibility of misunderstanding of some items, the lack of introspection of some participants and that of social desirability. Thus, the inclusion of information from parents and/or teachers could minimize the source of this error. In addition, measures of neuroimaging could add to the information gathered about cognitive functions. Finally, the cross-sectional nature of the study precludes the interpretation of neurocognitive performance; thus, it was not possible to establish whether differences in neurobehavioral assessment preceded or postdated the onset of suicidal difficulties. Future longitudinal follow-up studies could expand the understanding and correlation of neurocognitive functions and mental problems.

Despite the noted limitations and areas that would benefit from future research, the present study adds more information about the specific and relevant relationship between wellbeing and neurocognitive functions during adolescence. By comparing risk and nonrisk groups of adolescents, the present study contributes to information that could help to understand the underlying etiology of problems in subjective wellbeing and, thus, psychological difficulties in adolescents. Future studies could continue the study of phenotypic measures of cognitive domains with positive indicators of mental health such as wellbeing, prosocial behaviors, emotional regulation, self-esteem, as well as specifically combining these aspects with neuroimaging data, genomic parameters, and clinical assessment.

## Figures and Tables

**Table 1:** Descriptive statistics for the total sample and the risk and no-risk groups for PWI–SC.

Neurobehavioral Domain	Nonrisk		High Risk		Total Sample	
	*M*	*SD*	*M*	*SD*	*M*	*SD*
**Accuracy**						
Executive function	0.64	1.50	0.25	1.85	0.45	1.69
Episodic memory	1.24	1.63	0.33	1.90	0.79	1.82
Complex cognition	1.00	1.98	0.15	2.35	0.58	2.21
Social cognition	1.90	1.09	−0.02	2.35	0.94	2.07
**Speed domains**						
Executive function	−0.51	1.92	0.09	1.97	−0.21	1.96
Episodic memory	0.02	2.17	0.09	2.11	0.06	2.13
Complex cognition	0.19	2.00	−0.43	1.95	−0.12	1.99
Social cognition	−0.10	2.09	−0.58	1.92	−0.34	2.01
Sensorimotor	−0.09	1.16	−0.28	0.97	−0.18	1.07
**Efficiency**						
Executive function	0.13	2.04	0.34	2.40	0.24	2.22
Episodic memory	1.26	2.59	0.43	3.00	0.85	2.83
Complex cognition	1.19	3.27	−0.28	3.69	0.46	3.55
Social cognition	1.80	2.54	−0.60	3.21	0.60	3.12

**Table 2:** Accuracy neurocognitive performance scores by group.

Neurobehavioral Domain	Group				*p*	*Partial* η^2^
	Nonrisk		High-Risk			
	*M*	*SE*	*M*	*SE*		
Executive function	0.64	0.18	0.25	0.18	≤0.05	0.037
Episodic memory	1.24	0.19	0.33	0.19	>0.05	0.003
Complex cognition	1.00	0.23	0.15	0.23	≤0.05	0.041
Social cognition	1.90	0.20	−0.02	0.20	>0.05	0.001

**Table 3:** Speed neurocognitive performance scores by group.

Neurobehavioral Domain	Group				*p*	*Partial* η^2^
	Nonrisk		High-Risk			
	*M*	*SE*	*M*	*SE*		
Executive function	−0.43	0.21	0.09	0.21	>0.05	0.003
Episodic memory	0.08	0.24	0.09	0.23	≤0.05	0.032
Complex cognition	0.23	0.22	−0.43	0.22	≤0.05	0.069
Social cognition	−0.06	0.22	−0.06	0.22	≤0.05	0.052
Sensorimotor	0.37	0.22	0.03	0.22	>0.05	0.002

**Table 4:** Efficiency neurocognitive performance scores by group.

Neurobehavioral Domain	Group				*p*	*Partial* η^2^
	Nonrisk		High-Risk			
	*M*	*SE*	*M*	*SE*		
Executive function	0.67	0.25	−0.50	0.26	≤0.05	0.061
Episodic memory	1.18	0.33	−0.02	0.33	≤0.05	0.016
Complex cognition	1.38	0.32	−0.48	0.33	≤0.05	0.072
Social cognition	1.00	0.34	0.05	0.35	≤0.05	0.018
